# Applying Behavioral Biometrics to Mobile Device Use Measurement in Children: Evaluating the Impact of Training Data Size, Proximity, and Type on Model Performance

**DOI:** 10.1007/s41347-025-00537-8

**Published:** 2025-07-07

**Authors:** Olivia L. Finnegan, Hongpeng Yang, Bridget Armstrong, Srihari Nelakuditi, Rahul Ghosal, James W. White, Aliye B. Cepni, Zifei Zhong, Yan Tong, Michael W. Beets, Elizabeth L. Adams, Sarah Burkart, Erik A. Willis, R. Glenn Weaver

**Affiliations:** 1 Department of Exercise Science, University of South Carolina, 921 Assembly Street, Columbia, SC 29201, USA; 2 Department of Computer Science and Engineering, University of South Carolina, 301 Main Street, Columbia, SC 29208, USA; 3 Department of Epidemiology and Biostatistics, University of South Carolina, 915 Greene Street, Columbia, SC 29201, USA; 4 Center for Health Promotion and Disease Prevention, University of North Carolina Chapel Hill, 1700 M.L.K. Jr Blvd #7426, Chapel Hill, NC 27514, USA

**Keywords:** Mobile device use, Digital media use, Passive sensing, Screen time

## Abstract

**Objective:**

Passive sensing applications are limited by their inability to determine who is using a device, a critical concern in child mobile device use research, where devices are often shared between siblings or between a child and their parent. Our previous work leveraged behavioral biometrics to identify a target child user; however, it is unknown what type of training data is necessary for optimal model performance. This study evaluated model performance across different characteristics of training data.

**Methods:**

Thirty-six children (11.3 ± 0.9 years, 56% female) self-selected a video or a game on iPads for 10 min while laying and for another 5 min while sitting. The SensorLog application captured iPad accelerometer and gyroscope data while the child interacted with the device. Machine learning algorithms including Neural Network (NN), Random Forest (RF), k-Nearest Neighbors (k-NN), and SwipeFormer were applied to determine the most important aspects of training data to optimize model performance. The aspects of training data evaluated included (1) varying the length (i.e., seconds of training data), (2) varying the user position (i.e., sitting, laying), and (3) varying the time proximity between training and testing data. F1 score was used to evaluate model performance.

**Results:**

The SwipeFormer F1 scores were lowest when the training data was further from the test data (0 when training data was 11 min away from test data) and highest when training data was close to test data (0.91 when training data was the minute preceding test data). The SwipeFormer F1 scores were highest when predicting the user laying from laying (0.97) and sitting from sitting (0.94), and lowest when predicting the user sitting from laying (0) and laying from sitting (0). The length of training data had little impact on performance, with a SwipeFormer F1 score of 0.91 when training on one minute of data and a SwipeFormer F1 score of 0.94 when training on twelve minutes of data.

**Discussion:**

Because researchers would likely be predicting users at different timepoints than their training data, research should focus on improving model performance for identifying users independent of time proximity for training and test data.

## Introduction

Current evidence on the impact of mobile device use on children’s health and development is highly reliant on self- and parent-report measures (Domoff et al., 2019a, b; Girela-Serrano et al., 2022). However, subjective reports of mobile device use are problematic due to recall and social desirability bias, (Kabali et al., 2015) in addition to their insufficient ability to capture short bouts of device engagement (Radesky et al., 2020). Children also use mobile devices independently of their parents, (Domoff et al., 2019a, b) further limiting parent-report estimates. Given the on-demand and portable nature of mobile devices, objective measures are necessary to more precisely measure time spent on such devices (Byrne et al., 2021).

Passive sensing applications are an emerging solution to address the limitations of subjective reports of mobile device use (Domoff et al., 2021). Beyond timing and frequency of device usage, these applications also provide data on what types of applications and content are being used on mobile devices (Barr et al., 2020). While passive sensing presents a novel and important step forward for the field of mobile device use measurement, a critical limitation of these applications is that they do not provide an indicator of who is using the device (Byrne et al., 2021; Milkovich & Madigan, 2020; Radesky et al., 2020). Shared mobile devices are increasingly common in households with children, and children frequently share mobile devices with siblings and/or parents (Danet et al., 2022; Radesky et al., 2020). In a recent sample, 70.6% of Android users and 61.8% of iOS users shared mobile devices across their family with young children (Radesky et al., 2020). On average, children acquire their first independent mobile phone between 11 and 12 years of age (Sun et al., 2023). Therefore, within the field of child media measurement, capturing the ***user*** of the device is critical.

Recent research has highlighted the potential of applying behavioral biometrics, an area of research within the field of cybersecurity, to address this problem (Finnegan et al., 2024a, b; Finnegan et al., 2024a, b). Behavioral biometrics uses data derived from mobile device sensors to authenticate device users based on their unique way of interacting with the device (Meng et al., 2015). The accelerometer, gyroscope, and touch sensors are commonly used within the field of behavioral biometrics to authenticate users (Finnegan et al., 2024a, b). This approach is in contrast to physiological biometric authentication, which uses static traits of a user (i.e., fingerprint, facial recognition) to authenticate the device user. Behavioral biometrics offer several advantages over physiological biometrics, including privacy preservation and the capacity for continuous authentication. Physiological biometrics necessitates more user participation and has therefore been used for authentication only at initial login (Meng et al., 2015). Behavioral biometric authentication may be more appropriate for application to this context, as the sensors leveraged (i.e., motion sensors) are more accessible and feasible to use than the sensors used for physiological biometric authentication (i.e., camera). Within this public health context, motion sensors (accelerometer, gyroscope) have been used to detect a target child in a sample of nine children (Finnegan et al., 2024a, b). Model performance was strong in that proof-of-concept study, yielding F1 scores above 0.9, suggesting the potential of applying behavioral biometrics to address the limitations of passive sensing applications.

While behavioral biometrics could be used to augment passive sensing for more accurate mobile device use measurement, there is a limited understanding of the optimal conditions for application. For instance, behavioral biometrics largely uses machine learning (Abdulhak & Abdulaziz, 2018; Alzubaidi & Kalita, 2016), in which models are first trained with a training dataset, and subsequently tested with a separate testing dataset (Uçar et al., 2020). Thus, a key first step prior to applying behavioral biometrics to a public health context is to understand what type of training data researchers need to gather from participants to optimize model performance. More specifically, it is critical to determine how different or similar the training data needs to be to the testing dataset that is used for prediction. Therefore, the purpose of this study is to examine model performance across different types of training data. These findings will inform the type of training data needed to use behavioral biometric authentication in a public health research context.

## Methods

### Protocol

Data for the current study was collected opportunistically from the semi-structured physical activity protocol of the Wearables for Kids (W4K) study, which aimed to develop and validate equations to estimate children’s physical activity energy expenditure using consumer wearables. The W4K study, described elsewhere (Weaver et al., 2023), used a 60-min semi-structured physical activity protocol, with activities ranging from sedentary to vigorous intensity (Supplementary Table 1). The current study leveraged the two-part sedentary portion of this protocol, where children were laying (10 min) and then subsequently sat in a chair (5 min), all while interacting with an iPad. Children were allowed to self-select a YouTube video or a game on the iPad (i.e., Candy Crush, Subway Surfers, Crossy Road) during both sedentary activities. During the protocol, research assistants directly observed active iPad use of the child and recorded the start and end time of each sedentary activity to the 1-s level. The University of South Carolina Institutional Review Board approved study procedures (Pro00116257).

### Sample

The current study included 36 children from the W4K study who were between the ages of 5 and 12 years and were recruited from one southeastern metropolitan city through afterschool programs, summer day camps, newsletters, social media, and referrals. To be eligible for the W4K study, children had to be able to be physically active without an assistive device. Children were not eligible if they had a neuro-muscular disease.

### Motion Sensors & Devices Used

The open-source sensor tracking iOS application, Sensor-Log, was installed on each iPad. This application was used to continuously record motion sensor output during the sedentary portion of the W4K protocol. Details on this application are freely available (Thomas, n.d.) Briefly, SensorLog can access and record data streams on iOS devices at a sampling rate of up to 100 Hz. SensorLog can capture a variety of sensor streams, including raw accelerometer and gyroscope data, which were included in this study. Research assistants configured the settings on the SensorLog application to be set to 100 Hz for both the raw accelerometer data (X, Y, Z axes in gravity) and gyroscope data (X, Y, Z axes in radians/second). The accelerometer and gyroscope are physical sensors built into most modern mobile devices and these were selected as the data streams given their widespread application in behavioral biometric research (Corpus et al., 2016; Li & Bours, 2018).

This study used iPads (9th generation with iOS 16.5.1) for several reasons. First, iPads are mobile devices that have built-in motion sensors that have been applied in past behavioral biometric research (i.e., acceleration, gyroscope, and orientation) (Davis et al., 2020). Additionally, most previous behavioral biometric research has used devices with the Android operating system, which is a limitation given the widespread use of iOS devices (Finnegan et al., 2024a, b). Using iPads, which operate on the iOS operating system, may fill a gap for a relatively large share of the tablet market. Additionally, the use of an open-source sensor tracking application may make this approach generalizable to Android devices. The selection of a tablet, rather than a smartphone, was supported by evidence that tablets are highly used by children, particularly among the ages of this study’s sample (*The Common Sense Census: Media Use by Kids Age Zero to Eight*, 2017).

### Data Pre-Processing & Processing

Data were exported from iPads as a CSV file and imported into Python code for data processing and analysis. For time, the variable titled “accelerometerTimestamp_sinceReboot”, from the SensorLog application, was selected as opposed to the timestamp generated by SensorLog (loggingTime(txt) data). The accelerometerTimestamp_sinceReboot time was used because buffering and operating system specific delays caused duplicate readings in the loggingTime(txt) variable. Data were aggregated to the 1-s level with feature vectors calculated for each non-overlapped time window. A min–max normalization technique was applied, using a Scikit Learn package in Python (*MinMax Scaler*, n.d.).

### Feature Selection

Previous work has identified a feature set of 56 features that are salient for distinguishing a target user in a sample of children (Finnegan et al., 2024a, b; Finnegan et al., 2024a, b). These features include the mean, maximum, minimum, root mean square, variance, standard deviation, skewness and kurtosis of yaw, pitch, roll, acceleration (X, Y, Z axes), and vector magnitude. The complete feature set list is presented in Supplementary Table 2. Yaw, pitch, and roll are features generated by the accelerometer and gyroscope and were calculated from the sensor output using freely available software (*Motion Sensor*, n.d.) Yaw is rotation about the Y axis, pitch is rotation about the X axis, and roll is rotation about the Z axis. Vector magnitude is the square root of the sum of the squares of the X, Y, and Z axes. These motion features have been demonstrated to be effective in identifying unique users, as they can differentiate between the unique movement patterns among individuals (Cheng et al., 2020).

### Analysis: Machine Learning Approaches

The field of behavioral biometrics largely uses machine learning (Abdulhak & Abdulaziz, 2018; Alzubaidi & Kalita, 2016), which is a type of artificial intelligence that broadly aims to develop algorithms and models for classification and prediction. Machine learning trains models and algorithms to make predictions and learn patterns through training data. While machine learning *algorithms* are the techniques used for learning from the patterns of the data, machine learning *models* are the learned representation of the patterns of the data, which can then make predictions.

Consistent with previous research applying behavioral biometrics to the context of mobile device use measurement, four popular supervised machine learning models were used (Davis et al., 2020; Lamiche et al., 2019; Maghsoudi & Tappert, 2016; Putri et al., 2016; Smith-Creasey & Rajarajan, 2019; Zaidi et al., 2022). These algorithms include Neural Network (NN), k-Nearest Neighbors (k-NN), Random Forest (RF), and SwipeFormer. A neural network (NN) is a feed-forward architecture comprising multiple layers of nodes (neurons), where information flows in one direction from the input layer to the output layer. Each node processes the input from the previous layer by applying a weighted sum and an activation function. Through iterative training and weight adjustments, the network learns to distinguish between multiple classes, improving its predictions over time. k-NN is a machine learning method used for both classification and regression, which relies on the proximity of data points. It identifies the k nearest neighbors from the training data for a new data point and makes predictions based on the similarity of these neighbors. RF is a popular machine learning algorithm that fits several decision tree classifiers on sub-samples of the dataset and averages to optimize predictive accuracy. This functionality helps to reduce the threat of overfitting. In addition to these conventional machine learning models, we also incorporated SwipeFormer, a recently proposed Transformer-based deep learning architecture for mobile behavioral biometrics (Delgado-Santos et al., 2024). SwipeFormer is a deep learning technique that consists of two modules: (1) a transformer-based feature extractor and (2) a similarity computation module (Delgado-Santos et al., 2024). Transformer-based approaches are becoming highly used in behavioral biometrics given their strengths in user authentication using rich spatiotemporal data like mobile device data (Guo et al., 2024; Hu et al., 2023). Additionally, transformer-based approaches can improve continuous authentication, as it overcomes the limitations of other approaches that rely on static features (Guo et al., 2024). In our implementation, SwipeFormer was applied to raw sensor data, including yaw, pitch, roll, and acceleration across X, Y, and Z axes. This multimodal time-series input was processed through SwipeFormer’s dual-stream architecture, temporal, and frequency modules. Each stream has a self-attention mechanism and convolutional layers, facilitating the extraction of discriminative biometric features. To enable end-to-end classification, the final feature representation was passed through a multilayer perceptron (MLP) to output the prediction. All models used in this study have previously been applied in biometric authentication literature (Davis et al., 2020; Delgado-Santos et al., 2024; Lamiche et al., 2019; Maghsoudi & Tappert, 2016; Putri et al., 2016; Smith-Creasey & Rajarajan, 2019; Zaidi et al., 2022).

In machine learning, there are two important phases in developing and evaluating the model. First, there is the Training Phase, where the model is fed the *training* dataset, which includes the selected features and corresponding labels. In this phase, the model learns how to best use the features to predict the desired outcome. Then, there is the Testing Phase, when model performance is evaluated with a separate *testing* dataset. For this study, all machine learning models were cross validated using an 80/20 approach, where 80% of the data was used for training the model and the remaining 20% was used for validation of the model. The machine learning analyses were specific to each research question, which are outlined below.

This study focused on three characteristics of training data and evaluated the impact of modifying these characteristics on model performance. The conditions applied in this study included: (1) implementing unseen negative samples (i.e., unknown individuals not in the training data but in the testing data), (2) varying the length (i.e., seconds), (3) varying the time proximity between training and testing data, and (4) varying the user position (i.e., sitting, laying). The exclusion of samples in the training data that appear in the testing data would better simulate how data collection would operate in a real-word public health context. Researchers may not be able to get training data from siblings or parents of the target child, and it is necessary to evaluate model performance when unknown individuals (i.e., individuals not in the training data) interact with the shared device. Additionally, understanding the length of training time necessary for optimal performance is critical information for researchers developing protocols to gather training data. Specifically, it’s useful to develop protocols that gather sufficient amounts of training data, but also optimize time and resource constraints. Lastly, understanding how similar the training data needs to be to the testing data in terms of user position is another important factor in protocol development for gathering training data.

The current work applies motion sensor authentication, from the field of behavioral biometrics to a novel context (i.e., child mobile device use measurement), specifically using per-user models. This per-user modeling is similar to an authentication approach used in cybersecurity. For this per-user approach, researchers would gather training data specific to the target child that they will identify, and models would be created that are specific to each target child. Researchers would additionally need to gather training data over time, as physiological changes in children over time may affect the performance of behavioral biometrics approaches.

The analysis used to test each research question (RQ) is provided here:

Evaluate if a machine learning model can identify a target child when tested on out of sample data (i.e., testing dataset includes unknown participant data).

– RQ #1

To address the first research question, machine learning models were trained in a multi-step process. First, four children were taken out of the sample pool as unseen negative samples, called “decoys”. Second, for the other 32 participants, each participant served as a target child in their own machine learning model. Each of the models were trained on the data from the target child (1 of 32) and the other 31 children (without the four decoys). This training data included one random minute of “sitting” and one random minute of “laying” from each of the 32 participants. We used a random minute of “laying” and “sitting” data to ensure that both positions were included in the training data and because this research question was not evaluating the impact of the location of the training data with respect to the testing data. This research question was also not focused on the amount of training data, which was why we chose a total of two minutes of training data. Third, the models were applied on the testing dataset, which included the target participant (1 of 32) and the four decoys. This testing data was comprised of the last 3 min of sitting. This was performed across all 32 children. This same approach of including unseen negative samples (i.e., decoys) in the test set was used for the subsequent analyses (RQ# 2–4). Supplementary Fig. 1 displays the training and testing data creation process for this decoy approach, where the models generated were per-user.

Identify the minimum amount of training data needed to identify a specific child.

– RQ #2

Identify the impact of time proximity between training and testing data.

– RQ #3

To address the second and third research questions, we conducted two different analyses. For both analyses, the data was split into training and testing, with the last two minutes of the sedentary protocol being the test set for all participants. For the first analysis, the training data started at 1 min and increased by 1 min iteratively (1, 2, 3, 4, 5, 6, 7, 8, 9, 10, 11, 12-min training sets), with the testing data remaining the same for each analysis (2 min of sitting). In the first analysis, the training data increased from the end of the training dataset (Fig. 2a). Therefore, as the training dataset continued to get larger in size, the training dataset also moved increasingly closer to the testing dataset. In the second analysis, the training data started at 12 min (9 min laying, 3 min sitting) and was reduced by 1 min iteratively, with the testing data remaining the same for each analysis (2 min of sitting). It is worth noting that the training data was reduced from the beginning of the training dataset in the second analysis (Fig. 2b). Therefore, even as the training dataset continued to get smaller in size, the distance between the training dataset and the testing dataset remained the same. Model performance was evaluated at each minute length of training data (12, 11, 10, 9, 8, 7, 6, 5, 4, 3, 2, and 1-min training sets) across both analyses. Similar to RQ #1, this was run across all 32 participants, with the four decoys held out of the training dataset but included in the testing dataset.

Evaluate the impact of type of training data (e.g., sitting or laying) on the performance of the model in distinguishing the target child.

– RQ #4

To address the fourth research question, we conducted six different analyses. First, we evaluated how models performed in identifying the user while laying when trained on similar laying data. For this analysis, models used training data that was 2 min laying, and then tested on 2 min laying. Second, we assessed if this also worked for sitting and we evaluated how models performed in identifying the user while sitting when trained on similar sitting data. To do this, models were trained on 2 min sitting, and then tested on 2 min sitting. Third, we wanted to assess if models could distinguish the user when trained on different data, therefore, we evaluated how models performed in identifying the user while sitting when trained on laying data. To do this, models were trained on 2 min laying, and then tested on 2 min sitting. Fourth, we reversed this procedure to evaluate how models performed in identifying the user while laying when trained on sitting data. To do this, models were trained on 2 min sitting, and then tested on 2 min laying. Fifth, we wanted to assess model performance when using a combination of similar and different data, therefore, we evaluated how models performed in identifying the user while laying from training on 1 min laying and 1 min sitting. To do this, models were trained on 1 min of sitting and 1 min of laying and tested on 2 min laying. Lastly, we flipped this procedure to evaluate model performance in identifying the user while sitting from training on 1 min laying and 1 min sitting. To do this, the models were trained on 1 min of sitting and 1 min of laying and tested on 2 min sitting.

We additionally evaluated model performance across each possible minute of training to address the fourth research question. For instance, in the first analysis where we trained on 2 min of laying and tested on 2 min of laying, we used each possible consecutive 2-min combination of laying data before the test laying data. This allowed us to evaluate how proximity to the test data impacted model performance. Each of the six analyses were conducted across all 32 participants, with the four decoys held out of the training dataset but included in the testing dataset. The decoys were held constant across analyses.

### Machine Learning Model Hyperparameters

Machine learning model training uses *hyperparameters*, which are external aspects specific to each algorithm (i.e., trees in the RF algorithm, layers of the NN algorithm). Determining the optimal hyper-parameters for each algorithm, known as hyperparameter tuning, is an iterative process based on the performance of the model. In the model training process, we used GridSearch cross-validation to determine the best hyper-parameters and set a threefold cross-validation on the training set. The parameter ranges included: parameter *k* = {5,10,100,200} for k-NN, the maximum depth of the tree *d* = {10,50,100} for RF, and the strength of the L2 regularization term *α* = {10^−1^, 10^−2^, …, 10^−5^} for NN.

### Evaluation Metrics

For each analysis, model performance was summarized across the 32 participants using the median and interquartile range (25th and 75th percentile). The median and interquartile range were used to summarize across participants as opposed to the mean and standard deviation because each evaluation metric is bounded at 1 and the metric performance is not normally distributed. Model performance was evaluated using four model evaluation metrics: F1 score, accuracy, precision, and recall. The *F1 score* is a measure of predictive performance and is a weighted score of the combined precision and recall. *Accuracy* measures the overall correctness of predictions made by a classification model [(true positives + true negatives)/all observations]. *Precision* is a metric of the positive predictive ability of a model, while *recall* measures the ability of a model to correctly identify positive instances. Each of these metrics range in value from 0 to 1, with 1 being the strongest performance, and 0 being the worst performance. The goal in model selection was to maximize each of these metrics.

## Results

Demographic characteristics of the opportunistic sample are presented in Table 1. Of the sample of 36 children participating in this study, 56% were female, 14% were Black and the mean age was 11.3 (SD = 0.9) years.

### Model Performance

Evaluate if a machine learning model can identify a target child when tested on out-of-sample data (i.e., the target child’s data is not included in training data).

– RQ #1

**Fig. 1:**
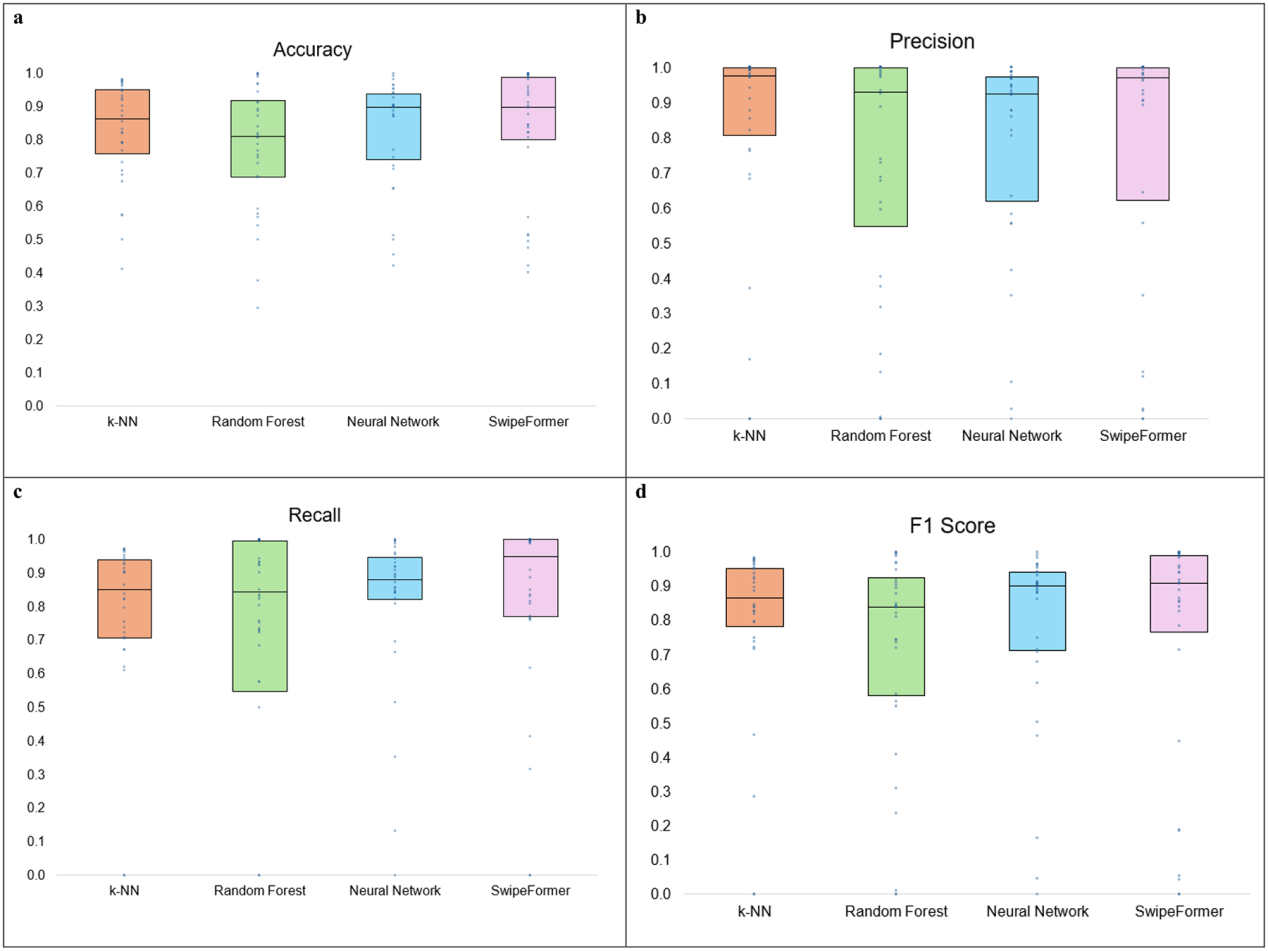
**a-d:** Performance Evaluation Metrics Across Models. k-NN: k-Nearest Neighbors

Figure 1a–d presents box plots of model performance (k-NN, RF, NN) across all evaluation metrics when unseen negative samples (i.e., “decoys”) were included in the testing data, but not in the training data. Model performance was comparable across all algorithms. The RF model had greater variability in metric performance when using precision, recall, and F1 score, compared to the other classifiers (k-NN, NN, SwipeFormer). Since SwipeFormer achieved the best performance in terms of F1 score in the first experiment, it will be used as the evaluation method for the next three experiments.

Identify the minimum amount of training data needed to identify a specific child.

– RQ #2

Identify the impact of time proximity between training and testing data.

– RQ #3

**Fig. 2:**
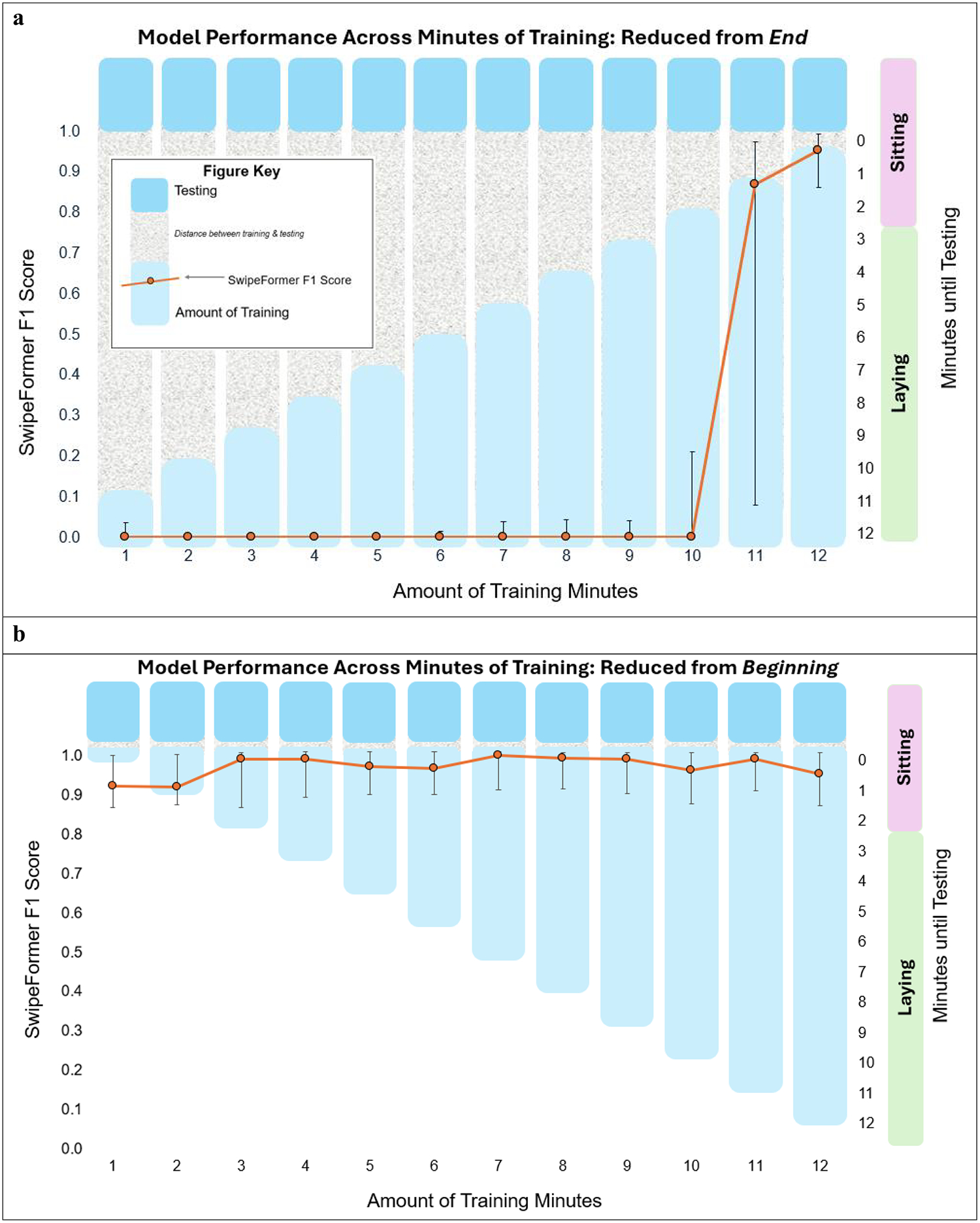
**a-b:** SwipeFormer F1 Score Across Minutes of Training. Presenting Median and Interquartile Range (25th and 75th Percentile). The Right Y Axis Represents Both: (1) The Minute(s) of the Protocol Used for Testing (of the 10 Minutes Laying, 5 Minutes Seated), and (2) The Proximity of the Training Set to the Test Set (i.e., Minutes Until Testing)

SwipeFormer F1 score performance across amount of training is presented in Fig. 2a–b. Figure 2a presents the model performance when the training minutes are building from the end (i.e., the training set gets closer to the testing set). Figure 2b presents model performance when the training minutes are reduced from the beginning (i.e., the training set always remains close to the testing set). Model performance is higher the closer the training set is to the testing set (Fig. 2a). When the training set remains close to the testing set across minutes of training, model performance remains relatively high across all minutes of training (Fig. 2b).

Evaluate the impact of type of training data (e.g., sitting or laying) on the performance of the model in distinguishing the target child.

– RQ #4

**Fig. 3:**
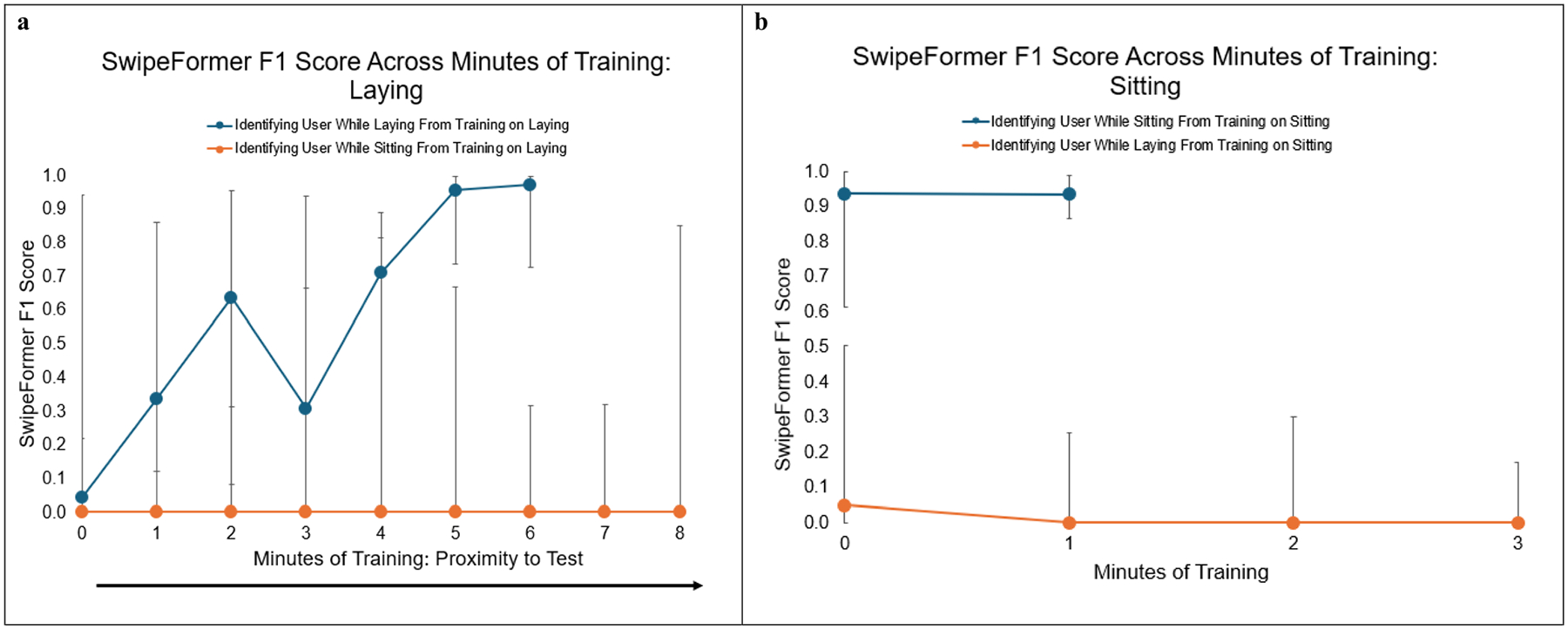
**a-b:** SwipeFormer F1 Score Across Different Types of Training Data. Presenting median and Interquartile Range (25th and 75th Percentile)

Figure 3a displays SwipeFormer F1 score performance when (1) identifying the target user when they are laying from training while they are laying (blue line), and (2) identifying the target user when they are sitting from training while they are laying (orange line), as the proximity to the test set increases. Model performance is higher when the testing set is the same ‘type’ as the training set (i.e., when training and testing are both on the laying down portion of the protocol). As indicated by Figs. 2a–b, 3a also highlights that as the training set gets closer in proximity to the testing set, model performance increases when the testing data is the same type as the training data.

Figure 3b presents SwipeFormer F1 score performance when (1) identifying the target user while they are sitting from training when they are sitting (blue line), and (2) identifying the target user while they are laying from training when they are sitting (orange line), as the proximity to the test set increases. As depicted by the length of the blue line, since there was less time spent sitting during the protocol (5 min as opposed to 10 min laying), there was less ability to test the relationship of different amounts of training data with model performance when training on the sitting data. Similar to Fig. 3a, b also indicates that model performance is stronger when the testing set is the same type as the training set (i.e., when training and testing are both on the sitting portion of the protocol).

**Fig. 4:**
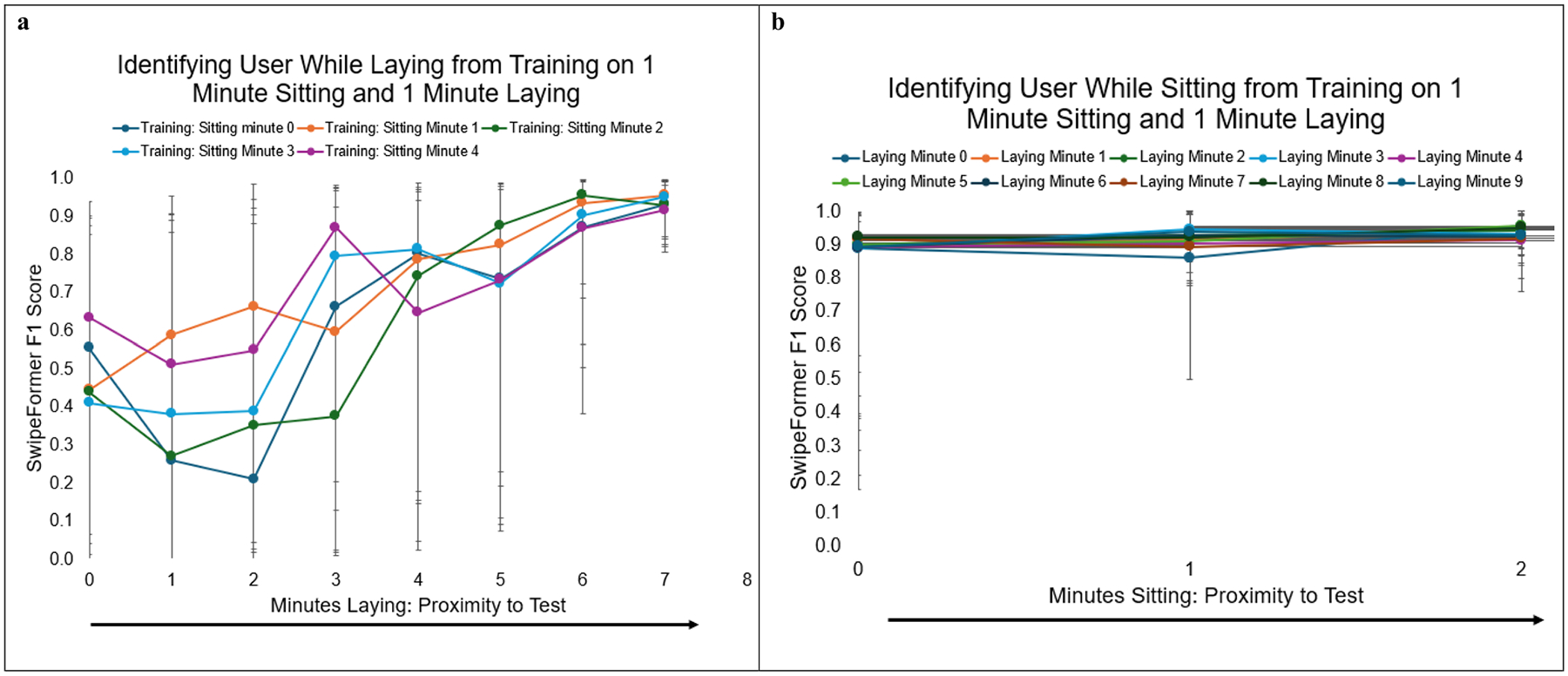
**a-b:** SwipeFormer F1 Score for Predicting User During Two Types of Activity from Training on 1-Minute Sitting and 1-Minute Laying. Presenting Median and Interquartile Range (25th and 75th Percentile)

SwipeFormer F1 score performance in identifying the target user while (1) laying and (2) sitting from training on 1-min sitting and 1-min laying is presented in Fig. 4a and b, respectively. Both graphs are shown across time, as the proximity to the test set increases. On Fig. 4a, which is identifying the target user while laying, each individual line represents a different training minute for *sitting*. On Fig. 4b, which is identifying the target user while sitting, each individual line represents a different training minute for *laying*. Similar to Figs. 3b, 4b is shorter in length due to the length of the sitting portion of the protocol. Figure 4a further emphasizes that proximity of the training set to the test impacts model performance, such that as the training set gets closer to the test set, model performance improves. For Fig. 4a, while the trend across minutes of laying indicates that proximity to test data improves performance, the minute of sitting does not appear to meaningfully impact performance. This further highlights the importance of the *type* of training data (i.e., user position), in conjunction to the *proximity* of training data to test data.

## Discussion

The purpose of this study was to evaluate the performance of machine learning models designed to predict the unique user with different types of training data. While previous research points to behavioral biometrics as a potential strategy to address the limitations of passive sensing applications (Finnegan et al., 2024a, b), the optimal conditions (i.e., training data characteristics) for application to a public health context are currently unclear. Such training data characteristics examined in this study included: (1) implementing unseen negative samples (i.e., unknown individuals not in the training data but in the testing data), (2) varying the length (i.e., seconds), (3) varying the time proximity between training and testing data, and (4) varying the user position (i.e., sitting, laying). Model performance was evaluated across each condition to better understand how to apply behavioral biometrics to mobile device use measurement in youth.

The study findings demonstrate that model performance is critically dependent on attributes of the training data. Model performance was greatly influenced by the ***position*** of the user (laying, sitting) during training data and the time ***proximity*** of the training data to the testing data. Interestingly, training data length did not appear to markedly impact model performance, rather, the proximity of the training data to the testing data appeared to be a more important factor. Even when training data and testing data were of the same position (laying, sitting), model performance improved as the training data got closer to the testing data. Therefore, the proximity of the training data to the testing data may be the most important factor in optimizing model performance. This is likely due to the similarities in user behavior when training data and testing data are close temporally. In a public health context, researchers would likely be predicting users at different timepoints than their training data (i.e., days or weeks later). Therefore, more research is necessary to optimize model performance when models are trained to predict users at future timepoints. In this analysis, we opted for a simpler model as a foundational approach to understand what training data characteristics are important for model performance. More complex and advanced approaches, such as long short-term memory (LSTM) models, may be effective in this context and could be applied in future research, as these models use long-term dependencies and sequences within the data to make predictions (Van Houdt et al., 2020).

Compared to the previous proof-of-concept study across nine participants (Finnegan et al., 2024a, b), model performance in this study was slightly lower. F1 scores in the proof-of-concept study for NN, k-NN, and RF were 0.92, 0.94, and 0.94, respectively. In the first analysis of the current study, F1 scores for NN, k-NN and RF were 0.90, 0.87, and 0.84, respectively. While both studies leveraged similar semi-structured physical activity protocols, used the same sensors (accelerometer, gyroscope), and applied the same machine learning algorithms, the current study applied the decoy technique to better simulate how this approach may operate in a real-word research context. Therefore, the discrepancy in model performance is likely due to including unseen negative samples in the testing data. This is an important finding for public health researchers looking to use this approach, as it more closely resembles real-world settings where children may be sharing mobile devices with parents or siblings who do not provide training data.

With machine learning analyses, a general understanding is that increased training size (i.e., greater number of samples for training) improves model performance (Uçar et al., 2020). The underlying principle of this assumption is that more training data provides more information for the model to accurately make predictions. This concept has also been demonstrated through previous research within the field of behavioral biometrics, where model performance improves with more training data (Buriro et al., 2017; Song et al., 2017; Syed et al., 2019; Yang et al., 2019). One study using accelerometer, orientation, magnetometer, gyroscope, and touch sensors evaluated model performance across 5, 10 and 15 samples of training data (Buriro et al., 2017). Model performance, determined through True Acceptance Rate and False Acceptance Rate, increased as the number of training samples increased. Another study using touch behavior examined model performance across different training set sizes ranging from 5 to 100 samples increasing by increments of 5 samples (Song et al., 2017). Equal Error Rate (EER) decreased markedly, indicating stronger model performance, as the training set size increased. Similarly, another study authenticating users through touch behavior compared model performance across training set lengths ranging from 20 to 160 samples, increasing in increments of 20 samples (Yang et al., 2019). Lastly, another study using touch gestures explored model performance across larger proportions of training data (10–100%), finding that EERs decreased as the training data percentage increased (Syed et al., 2019). Across these studies, model performance largely improved as training set length increased, further reinforcing the concept that model performance depends on the amount of training used in model development. These findings contrast with the current study, which found that training set size did not meaningfully impact model performance. This finding may be due to the shorter length (~ 15 min) and design of the protocol in the current study, which was completed in one “session”. This design is in contrast to other studies, which had multiple sessions and longer protocol times (Buriro et al., 2017; Song et al., 2017; Syed et al., 2019). Training length may be a more important factor for model performance when models are trained and tested across different sessions over time. Thus, future protocols should test this approach in multiple sessions and over longer periods of time.

The current study found that training data closer in proximity to the testing data produced higher performing models. There have been mixed findings in the behavioral biometric literature related to the proximity of training data to testing data (Ray et al., 2021). Contrary to the current study findings, a study using touch, keystroke, and motion sensors across two structured sessions evaluated both intra-session (training and testing data both from first session) and inter-session (training data from first session, testing data from second session) model performance (Ray et al., 2021). The average EER was slightly lower (7.9 vs. 8.3%), indicating better model performance, for the inter-session analysis. Their finding that model performance was slightly better in predicting the user at a future timepoint does not align with the current study. However, that study used touch behavior, which may be a more consistent behavior over time, whereas movement may be more variable and subject to situational differences. Another study using exclusively touch data compared model performance across different training and testing conditions: (1) when training and testing were in the same session, (2) when there was a gap of 10–12 min between the training and testing sessions and (3) when there was a gap of one week between the training and testing sessions (Roy et al., 2015). Similar to the current study, model performance worsened as the training and testing sessions grew further apart. However, model performance was strong across all training and testing conditions (EER ranging from 0 to 4%) (Frank et al., 2012; Roy et al., 2015), in contrast to the current findings that showed model performance was substantially worse as proximity of the training data was further from the testing data. This contrast in study findings may be due to the authors’ use of Hidden Markov Modeling (HMM), used in conjunction with the Viterbi algorithm, which uses temporal sequencing of the data over time to make predictions (Churbanov & Winters-Hilt, 2008). Conversely, the models tested herein were trained to predict the user at the second-level without data on the preceding seconds. As discussed previously, future research applying behavioral biometrics to this context should use modeling techniques that learn long-term patterns of the data (i.e., LSTM, HMM with Viterbi) to optimize model performance.

There is relatively limited research within the field of behavioral biometrics about how closely training data should resemble testing data (Smith-Creasey & Rajarajan, 2019; Syed et al., 2015, 2019). A study using gesture typing authentication, with a combination of touch and movement features, achieved lower EERs when both training and testing data were the same activity (i.e., both were sitting, both were walking, both were standing), as opposed to training on one activity and testing on another activity (Smith-Creasey & Rajarajan, 2019). Two other touch gesture studies examined how the posture of training and testing data impacted model performance (Syed et al., 2015, 2019). Posture in those studies was defined differently than the current study and was instead comprised of both device orientation (i.e., portrait or landscape) and device position (i.e., in hands, on table). Both studies found that keeping the posture consistent for both training and testing increased model performance. These findings are consistent with the current results, such that model performance is better when the training and testing data look similar. One potential reason for this is that movement of the iPad may be impacted by the orientation of the individual. Individuals may have different micro-movement behaviors when interacting with the iPad in different positions. Alternatively, another reason may be that models are overfit to identify the user by detecting the individual’s specific orientation rather than motion behavior patterns specific to the way the individual interacts with the device. Further research is necessary to uncover why performance was worse when training data and testing data are of different orientations. While the current study has emphasized the importance of gathering training data across both sitting and laying positions, these are somewhat contrived examples and further research is necessary to determine the optimal training necessary for predicting a user in free-living environments, across many positions and activities.

Although previous research within the field of cyber-security and behavioral biometrics have tested some of the same training data characteristics tested in the current study, the goal of the current study was to explore whether these findings are consistent in a novel research context. This study therefore is novel in presenting further exploration of using behavioral biometrics to improve objective mobile device use measurement. This study is additionally strengthened by applying a transformer-based approach, SwipeFormer, which are becoming highly used within biometric authentication because of their efficiency in parallel processing and ability to capture complex patterns common in mobile device use (Delgado-Santos et al., 2024). However, there are several limitations to consider. First, this study is limited by its reliance on opportunistic data collected from a protocol of a larger study. The protocol leveraged in this study was not specifically designed to test these research questions. The activity on the iPad (i.e., game or video) was not standardized, which could potentially impact model performance, as models may only be identifying the game being played. However, children used the same iPads with the same selection of games. Additionally, the laying portion of the protocol was for 10 min, while the sitting portion of the protocol was for 5 min. Future research should both standardize the game or video being played and use consistent time frames across different user positions and activities (i.e., sitting, laying, standing, walking).

Collectively, these findings highlight the importance of training data having the same *position* and close *proximity* to testing data for optimal model performance. In the context of applying this to public health, researchers must consider protocols that optimize resources and accommodate real-world settings, all while gathering the necessary training data for models to perform well on identifying the device user. While *length* of the training data does not appear to be an important factor for model performance, this study highlights the need for training data that appears similar to the testing data in terms of position (laying, sitting). In a practical sense, this would mean that researchers would need to gather data from similar positions to those in which they want to predict on. A training protocol may include children playing with an iPad while laying and sitting to improve model performance when predicting the user in those positions. Moreover, a more important factor related to model performance appears to be the proximity of the training data to the testing data. To better understand how behavioral biometrics would perform in a public health context, further research should prioritize improving model performance in identifying users at different timepoints than the training data.

## Supplementary Material

Supplementary Material

**Supplementary Information** The online version contains supplementary material available at https://doi.org/10.1007/s41347-025-00537-8.

## Figures and Tables

**Table 1:** Sociodemographics of Participating Children (N = 36)

**Sex**	**n (%)**
Female	20 (56%)
Male	16 (44%)
**Race**	
Black	5 (14%)
White	27 (75%)
Other; More than 1 race	2 (5.5%)
Unknown	2 (5.5%)
**Ethnicity**	
Not Hispanic or Latinx	33 (92%)
Hispanic or Latinx	3 (8%)
	**Mean (SD)**
**Age (yr)**	11.3 (0.9)

SD = Standard Deviation

## Data Availability

Data will be made available upon reasonable request from the authors.
