# Supplementary material for: Applying Behavioral Biometrics to Mobile Device Use Measurement in Children: Evaluating the Impact of Training Data Size, Proximity, and Type on Model Performance
Source: J Technol Behav Sci. Author manuscript; Available in PMC 2026 Jan 9. (PMC12782210; doi:10.1007/s41347-025-00537-8)
Supplement: Supplementary Material [file NIHMS2127369-supplement-Supplementary_Material.docx]

**Supplementary Material**

| **Supplementary Table 1. Wearables for Kids (W4K) Protocol** | | | |
| --- | --- | --- | --- |
| **Activity** | **Intensity** | **Description** | **Minutes Spent in Activity** |
| Resting, Lying down | Sedentary  ≤ 1.5 METs | Lying down in supine position - on a blanket on the ground; playing on iPad (playing games) | 10 |
| Video viewing | Sedentary  ≤ 1.5 METs | Sitting in a chair at a table playing on iPad (playing games). Instructed to minimize body movements | 5 |
| Walking casual | Light  >1.5 METs to < 3 METs | Walking self-selected pace casual with a research assistant | 5 |
| Walking Brisk | Moderate   ≥ 3.0 METs to < 6 METs | Walking self-selected pace brisk with a research assistant | 5 |
| Obstacle course | Vigorous   ≥ 6.0 METs | Participants will run through an obstacle course led by a research assistant | 5 |
| Break | Sedentary  ≤ 1.5 METs | Seated research assistant is describing next block | 3 |
| Highlighted portion denotes portion of the protocol used for the current study. | | | |

| **Supplementary Table 2.** List of All 56 Features |
| --- |
| **Feature Name** |
| Maximum yaw |
| Maximum roll |
| Mean yaw |
| Mean roll |
| Minimum roll |
| Minimum yaw |
| Mean acceleration along the Y axis |
| Minimum pitch |
| Maximum acceleration along the Y axis |
| Mean acceleration along the X axis |
| Mean acceleration along the Z axis |
| Maximum acceleration along the Z axis |
| Mean vector magnitude |
| Minimum acceleration along the Y axis |
| Mean pitch |
| Maximum acceleration along the X axis |
| Maximum pitch |
| Minimum acceleration along the X axis |
| Root mean square of pitch |
| Minimum acceleration along the Z axis |
| Variance of pitch |
| Standard deviation of roll |
| Standard deviation of acceleration along the Z axis |
| Root mean square of acceleration along the Z axis |
| Variance of acceleration along the Z axis |
| Standard deviation of yaw |
| Variance of roll |
| Root mean square of roll |
| Minimum vector magnitude |
| Standard deviation of acceleration along the X axis |
| Standard deviation of pitch |
| Maximum vector magnitude |
| Standard deviation of vector magnitude |
| Variance of yaw |
| Kurtosis of acceleration along the X axis |
| Skewness of vector magnitude |
| Standard deviation of acceleration along the Y axis |
| Root mean square of acceleration along the X axis |
| Kurtosis of acceleration along the Y axis |
| Root mean square of yaw |
| Root mean square of vector magnitude |
| Root mean square of acceleration along the Y axis |
| Variance of vector magnitude |
| Skewness of acceleration along the X axis |
| Kurtosis of yaw |
| Variance of acceleration along the Y axis |
| Variance of acceleration along the X axis |
| Kurtosis of acceleration along the Z axis |
| Kurtosis of vector magnitude |
| Skewness of yaw |
| Skewness of roll |
| Kurtosis of roll |
| Skewness of pitch |
| Skewness of acceleration along the Y axis |
| Skewness of acceleration along the Z axis |


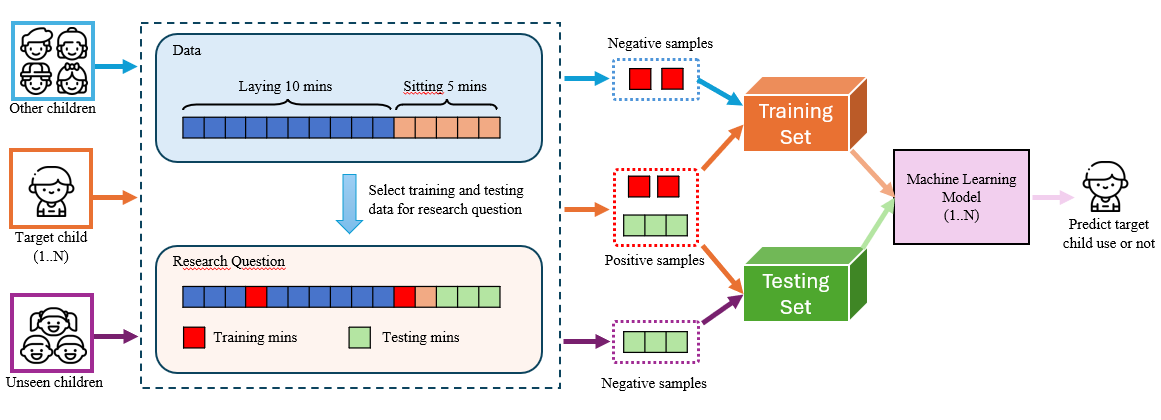


**Supplementary Figure 1.** Training and Testing Data Creation Process
